# Newspaper Coverage of Hospitals During a Prolonged Health Crisis: Longitudinal Mixed Methods Study

**DOI:** 10.2196/48134

**Published:** 2024-02-21

**Authors:** Frank van de Baan, Rachel Gifford, Dirk Ruwaard, Bram Fleuren, Daan Westra

**Affiliations:** 1 Department of Health Services Research Faculty of Health, Medicine and Life Sciences Maastricht University Maastricht Netherlands; 2 Department of Work and Social Psychology Faculty of Psychology and Neuroscience Maastricht University Maastricht Netherlands

**Keywords:** health communication, news coverage, media, misinformation, accuracy, news, reporting, newspaper, knowledge translation, COVID-19, dissemination, communication

## Abstract

**Background:**

It is important for health organizations to communicate with the public through newspapers during health crises. Although hospitals were a main source of information for the public during the COVID-19 pandemic, little is known about how this information was presented to the public through (web-based) newspaper articles.

**Objective:**

This study aims to examine newspaper reporting on the situation in hospitals during the first year of the COVID-19 pandemic in the Netherlands and to assess the degree to which the reporting in newspapers aligned with what occurred in practice.

**Methods:**

We used a mixed methods longitudinal design to compare internal data from all hospitals (n=5) located in one of the most heavily affected regions of the Netherlands with the information reported by a newspaper covering the same region. The internal data comprised 763 pages of crisis meeting documents and 635 minutes of video communications. A total of 14,401 newspaper articles were retrieved from the LexisNexis Academic (RELX Group) database, of which 194 (1.3%) articles were included for data analysis. For qualitative analysis, we used content and thematic analyses. For quantitative analysis, we used chi-square tests.

**Results:**

The content of the internal data was categorized into 12 themes: COVID-19 capacity; regular care capacity; regional, national, and international collaboration; human resources; well-being; public support; material resources; innovation; policies and protocols; finance; preparedness; and ethics. Compared with the internal documents, the newspaper articles focused significantly more on the themes COVID-19 capacity (*P*<.001), regular care capacity (*P*<.001), and public support (*P*<.001) during the first year of the pandemic, whereas they focused significantly less on the themes material resources (*P*=.004) and policies and protocols (*P*<.001). Differences in attention toward themes were mainly observed between the first and second waves of the pandemic and at the end of the third wave. For some themes, the attention in the newspaper articles preceded the attention given to these themes in the internal documents. Reporting was done through various forms, including diary articles written from the perspective of the hospital staff. No indication of the presence of misinformation was found in the newspaper articles.

**Conclusions:**

Throughout the first year of the pandemic, newspaper articles provided coverage on the situation of hospitals and experiences of staff. The focus on themes within newspaper articles compared with internal hospital data differed significantly for 5 (42%) of the 12 identified themes. The discrepancies between newspapers and hospitals in their focus on themes could be attributed to their gatekeeping roles. Both parties should be aware of their gatekeeping role and how this may affect information distribution. During health crises, newspapers can be a credible source of information for the public. The information can also be valuable for hospitals themselves, as it allows them to anticipate internal and external developments.

## Introduction

It is important for health organizations to communicate with the public during health crises [1-4]. Newspapers, both print and on the web, form an important mode of communication, given their relatively high perceived credibility, frequent use, and agenda-setting role [5-8]. Newspapers can inform the public of recent developments, including the extent of the crisis and threats, and preventive measures [3,9,10]. This enables the public to be well informed, can reduce fear, and stimulates the public to adopt the behavior needed for crisis mitigation [11-14]. However, communication can also induce public anxiety and make people refrain from appropriate behavior [14-16]; misinformation, an overabundance of information, or a wrongful depiction of certain issues or phenomena can be contributing factors [15,17,18]. Therefore, it is essential that both health organizations and newspapers facilitate the distribution of information that is accurate, timely, and valid [9,19,20].

Traditionally, governments and public health organizations have been the main organizations involved in communication during a health crisis [21-23]. However, the COVID-19 pandemic has been recognized by health communication scholars as the first large-scale health crisis to occur in a new media context, characterized by, among other elements, a deluge of information and misinformation [15,23,24], relatively high rates of skepticism toward media reporting [23,25], and new organizations involved in sharing information [23]. Hospitals emerged as such a new organization because of the central role they played during the crisis in ensuring the treatment of patients with COVID-19 [26-28], often being on the front pages of newspapers [29]. Furthermore, hospitals experienced firsthand the effects of misinformation and skepticism toward media reporting, including continued pressure on hospital functioning owing to low vaccination rates [30,31] and increased aggression toward hospital workers [32]. In light of the new media context and the negative effects associated with flawed communication, scholars have emphasized the importance of gaining a better understanding of information distribution through media [19,33].

Studies analyzing communication through newspapers so far have shown mixed results. Some studies have suggested adequate information distribution, for example, during the SARS-CoV-1 outbreak [34] or the H1N1 influenza pandemic [35,36]. However, other studies have been indecisive or deemed that communication could be improved, including those focusing on the West Nile virus outbreak [37], the H1N1 influenza pandemic [9,38,39], and the COVID-19 pandemic [8,40,41]. Although these studies provide valuable insights, some aspects remain understudied because most studies focused on epidemiological information [34-37,39] or included a short time frame (ie, <6 months) [8,40,41]. Furthermore, in light of the new media context, it remains unclear how information from new key stakeholders, such as hospitals, is being presented to the public. Overall, there is a need for further insights into how information from health organizations is presented to the public by newspapers during health crises [19,33,42]; therefore, scholars have specifically called for studies using both qualitative and quantitative components to understand this phenomenon [19].

Given this need, this paper presents a longitudinal mixed methods study examining the representation of hospital information in (web-based) newspaper reporting during 12 months of a health crisis in the Netherlands. Specifically, this study aimed to (1) explore the situation of hospitals during the first year of the COVID-19 pandemic, (2) determine the degree to which the representation of the identified themes in internal documents aligned with what was presented to the public in newspaper articles, (3) examine the extent to which hospital actors were included in newspaper articles reporting on hospitals, and (4) assess whether newspaper articles contained misinformation. The results of this study deepen our understanding of the portrayal of organizational information in the media and the role of both media and health care organizations in facilitating effective communication, especially during health crises [19]. Moreover, these insights can help optimize communication strategies and facilitate improved information distribution during a health crisis [19]. As such, this study contributes to health emergency preparedness [43].

## Methods

### Overview

This longitudinal mixed methods study compared internal hospital data with newspaper coverage over time using thematic, quantitative, and manifest content analyses. We focused on (web-based) newspaper reporting because in the Netherlands and many other European countries, both regional and national newspapers still form an important source of information for the public, especially during health crises [44]. This is contrary to some other countries, such as the United States or Argentina, possibly because of lower trust in newspaper reporting in these countries [44]. We focused on the first year of the pandemic to encapsulate the most pressing period in terms of hospital admissions and deaths within the Netherlands [45,46]. This study was part of a larger research project investigating hospitals’ responses to the COVID-19 crisis.

### Ethics Approval

Ethics approval was obtained for this study by the Faculty of Health, Medicine and Life Sciences Research Ethics Committee of Maastricht University (FHML-REC/2020/110).

### Setting

This study focused on the Dutch province of Limburg, one of the most heavily affected provinces in the country during the first year of the COVID-19 pandemic [47]. The province had 1,115,872 inhabitants as of January 1, 2021 [48]. During the first year of the pandemic (February 27, 2020, to February 28, 2021), the province experienced 71,348 confirmed COVID-19 cases and 1438 COVID-19–related deaths [46,49-53].

There are 5 hospitals located in the province of Limburg. These 5 hospitals vary in size, ranging from one of the smallest in the country to one of the largest in the country in terms of the number of available beds, the number of patients treated, and the number of staff. One of the hospitals is an academic hospital, whereas the other 4 are general hospitals, of which 2 are top clinical centers. Geographically, the hospitals are equally spread across the province. All included hospitals provide both inpatient and outpatient care and have a 24-hour emergency ward. All hospitals are members of the same regional acute care network; regional acute care networks are networks through which Dutch health care organizations coordinate crisis responses [54].

The province of Limburg has 1 major regional newspaper covering the entire province, namely De Limburger, with a daily circulation of >120,000 newspapers [55]. The newspaper has no specific political leaning and is considered neutral [56,57]. The newspaper is available both web-based and in print.

### Data Collection

This study used 2 data sources: internal data from all 5 hospitals and all related media coverage from the main newspaper of the province. On March 4, 2020, the province had the first confirmed COVID-19 case [58]; therefore, the time frame was set from March 1, 2020, to February 28, 2021.

Regarding the hospital data, each of the 5 hospitals provided us with in-depth organizational data from the first year of the COVID-19 pandemic in the Netherlands. Specifically, we were given crisis meeting minutes (action and decision lists) from all meetings held between March 1, 2020, and February 28, 2021, and internal communication videos posted during the same time frame. Ultimately, 763 pages of written documents were collected, together with 635 minutes of video communications. The documents and videos contained a variety of content, including detailed information on discussions held within the crisis teams, purchases made, and department-level issues.

The regional newspaper De Limburger was selected as a data source, as it is a large regional print newspaper, also available on the web, that covers the exact province where all the 5 hospitals are located. We included a regional newspaper as opposed to a national newspaper because, in light of gatekeeping theory, the regional newspaper would likely contain more articles about the hospitals [59,60]. We used the LexisNexis Academic (RELX Group) database to collect articles published in De Limburger. To retrieve relevant articles, the keywords used were as follows: “COVID-19,” “corona,” “care” (zorg), “hospitals” (ziekenhuis), or “hospitals” (ziekenhuizen). In total, 14,401 newspaper articles were identified and screened for relevance based on title and preview. Articles were deemed relevant when they included information on ≥1 of the 5 hospitals and contained information on anything related to COVID-19 in these hospitals. When in doubt of relevance, the articles were fully read and included if relevant. Of the 14,401 articles, 194 (1.3%) were included in the data analysis. The reasons for the exclusion of a large number of articles include the fact that many did not relate to COVID-19 or any of the hospitals. In part, this has to do with the fact that we used a broad search string (using “OR” instead of “AND”), as we did not want to miss any relevant articles. Furthermore, the keyword “care” (zorg) also means “ensure” in Dutch, leading to the inclusion of articles with this keyword but not related to COVID-19 and hospitals.

### Data Analysis

To assess newspaper reporting of the situation in hospitals, content and thematic analyses were performed [61,62]. All coding was done by hand, as opposed to computer-aided searches for certain terms, as this is more likely to lead to a more accurate interpretation of data [63].

Regarding the internal data (ie, the action and decision lists and communication videos), FvdB used thematic inductive coding to identify themes that characterized organizational reality during the pandemic [64]. This approach was deemed most suitable because through this technique, common themes revolving around a certain phenomenon (ie, the COVID-19 pandemic) can be identified [65]. It involved pattern recognition within the data, requiring constant careful rereading of the data and construction of themes based on the characteristics of the data [64]. The coding continued until no new themes emerged. Throughout the process, FvdB engaged in ongoing discussions with RG and DW regarding the emerging codes and identified themes and presented identified codes, grouped themes, and other updates. Through these discussions, the authors, for example, distilled *public support* as a main theme. This theme emerged through codes related to signs of public support and appreciation (eg, bringing gifts, care bonuses, and support through social media) and codes related to public aggression and frustration (eg, verbal assault of employees, impatient patients, and downplay of the severity of the crisis on social media). In total, 12 main themes were identified, which were used to create a codebook. Multimedia Appendix 1 includes the codebook with the definition of each theme and examples of quotes. This study was part of a larger project aimed at identifying hospitals’ challenges during the COVID-19 pandemic and related responses. For the project, interviews were conducted by FvdB and RG with hospital employees from all layers of the organization (eg, medical staff, managers, and chief executive officers [66,67]). This provided a certain safeguard that the main themes identified through the coding resonated with what was experienced in practice.

Quantitative thematic content analysis was used to determine the degree to which the representation of themes in the internal documents aligned with that in the newspaper [61]. The created codebook was used to deductively code the 194 newspaper articles and 763 pages of internal documents. For each newspaper article, sentences, paragraphs, or passages that encapsulated a certain theme from the codebook were coded [64]. For example, in one of the articles, the code human resources was assigned to the following paragraph:

Bottleneck in all hospitals remains the availability of people. Who also drop out. “Absenteeism is high,” says the hospital spokesperson. Many employees have [been] infected, many more than in the first wave. Moreover, the second wave lasts longer than the first.

Articles not covering any of the themes from the developed codebooks were assigned the code “miscellaneous” (6/194, 3.1%). If an article covered multiple themes, it was given multiple codes. The same approach was used for the internal documents (ie, each hospital’s action and decision lists per day). That is, sentences, paragraphs, or passages were deductively coded. For example, in one of the documents, the code ethics was assigned to the following passage:

When the corona law takes effect on Dec. 1, wearing a mouth mask will be mandatory in all public places. ...[But] we will not enforce this law because we have a care duty.

FvdB, RG, and DW engaged in initial deductive coding of the newspaper articles and internal documents. Differences in coding were discussed to reach a consensus on the coding procedure. FvdB coded the rest of the data and consulted with RG and DW in case of doubt. After the proportions of themes for the internal documents and newspaper articles were identified, chi-square tests were conducted to calculate differences between theme proportions of the internal documents and newspaper articles. Chi-square tests were performed using SPSS Statistics for Windows (version 27; IBM Corp). *P* values <.01 were considered statistically significant.

To assess the presence of misinformation in the newspaper articles, manifest content analysis was performed, which is a form of qualitative content analysis [62,68]. This method of content analysis regards the direct examination of text with a low level of abstraction and a low degree of interpretation [68]. This was considered an appropriate approach because we wanted to directly compare the text in newspaper articles (eg, statements on absenteeism rates, intensive care unit capacity, or change in visitor policies) with the text of the internal documents to assess whether the information in the newspaper articles aligned with the information in the internal documents.

## Results

### Identified Themes and Quantitative Differences in Coverage

The internal data revealed 12 themes regarding hospitals’ situation during the first year of the COVID-19 pandemic. The themes included COVID-19 capacity; regular care capacity; regional, national, and international collaboration; human resources; well-being; public support; material resources; innovation; policies and protocols; finance; preparedness; and ethics (refer to Multimedia Appendix 1 for a full description of themes and exemplary quotes). Table 1 shows the number of times the code of a theme was found in the internal documents and newspaper articles, the proportions of themes compared with the total amount of codes, and whether the proportions of the internal documents differed significantly from those of the newspaper articles. As can be seen, over the course of the year, the newspaper articles covered significantly more about COVID-19 capacity (*P*<.001), regular care capacity (*P*<.001), and public support (*P*<.001). The articles covered significantly less about material resources (*P*=.004) and policies and protocols (*P*<.001). No significant differences were found in the other themes.

**Table 1:** Comparison of the proportions of codes from the internal documents of the 5 hospitals with those from the 194 newspaper articles published during the first year of the COVID-19 pandemic in the Netherlands (March 1, 2020, to February 28, 2021).

Theme	Codes in internal documents (n=1437), n (%)	Codes in newspaper articles (n=375), n (%)	*P* value
COVID-19 capacity	186 (12.9)	78 (20.8)	<.001
Regular care capacity	133 (9.3)	63 (16.8)	<.001
Regional, national, and international collaboration	133 (9.3)	27 (7.2)	.32
Human resources	237 (16.5)	50 (13.3)	.21
Well-being	105 (7.3)	27 (7.2)	.86
Public support	20 (1.4)	22 (5.9)	<.001
Material resources	118 (8.2)	13 (3.5)	.004
Innovation	59 (4.1)	13 (3.5)	.79
Policies and protocols	276 (19.2)	40 (10.7)	<.001
Finance	54 (3.7)	13 (3.4)	.97
Preparedness	92 (6.4)	19 (5)	.49
Ethics	24 (1.7)	10 (2.7)	.01

### Changes in Reporting Over Time

**Figure 1:**
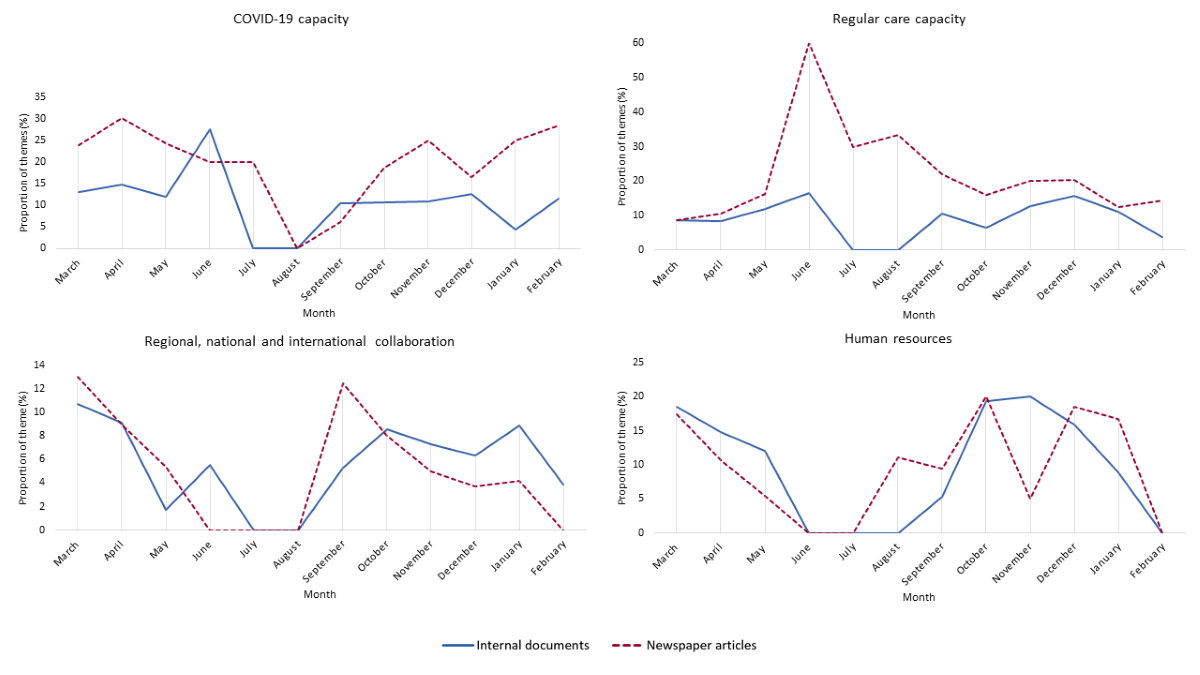
The distribution of theme proportions per month for the hospital documents and newspaper articles during the first year of the COVID-19 pandemic in the Netherlands (March 1, 2020, to February 28, 2021).

**Figure 2:**
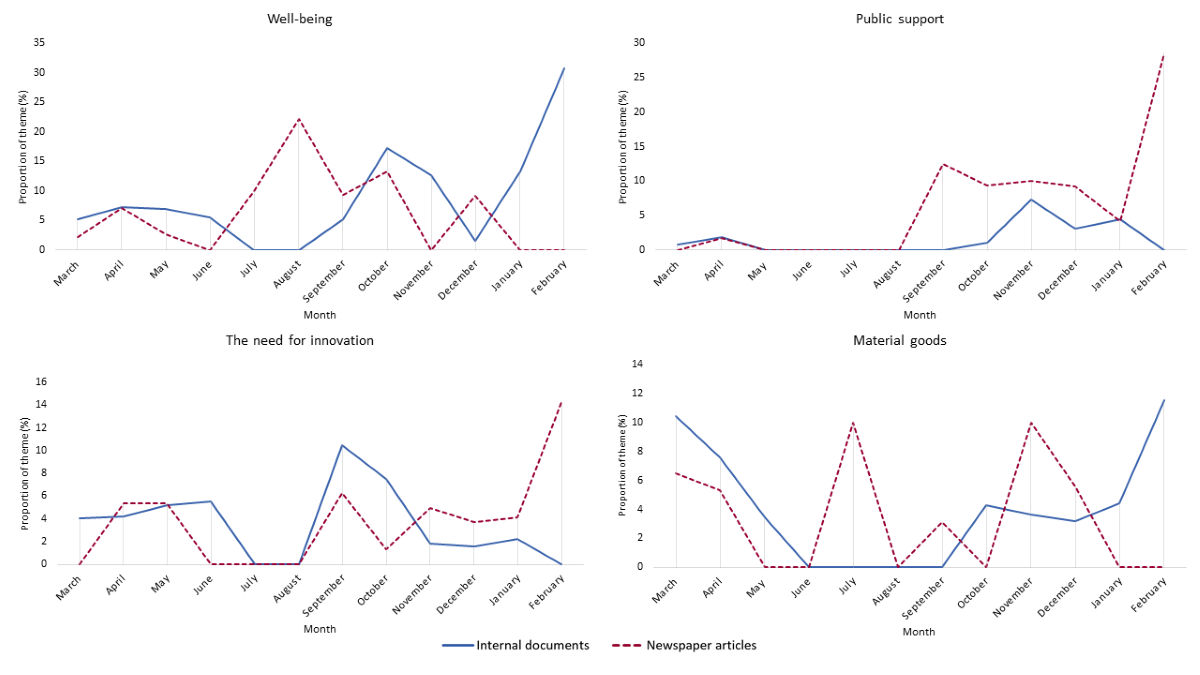
The distribution of theme proportions per month for the hospital documents and newspaper articles during the first year of the COVID-19 pandemic in the Netherlands (March 1, 2020, to February 28, 2021).

**Figure 3:**
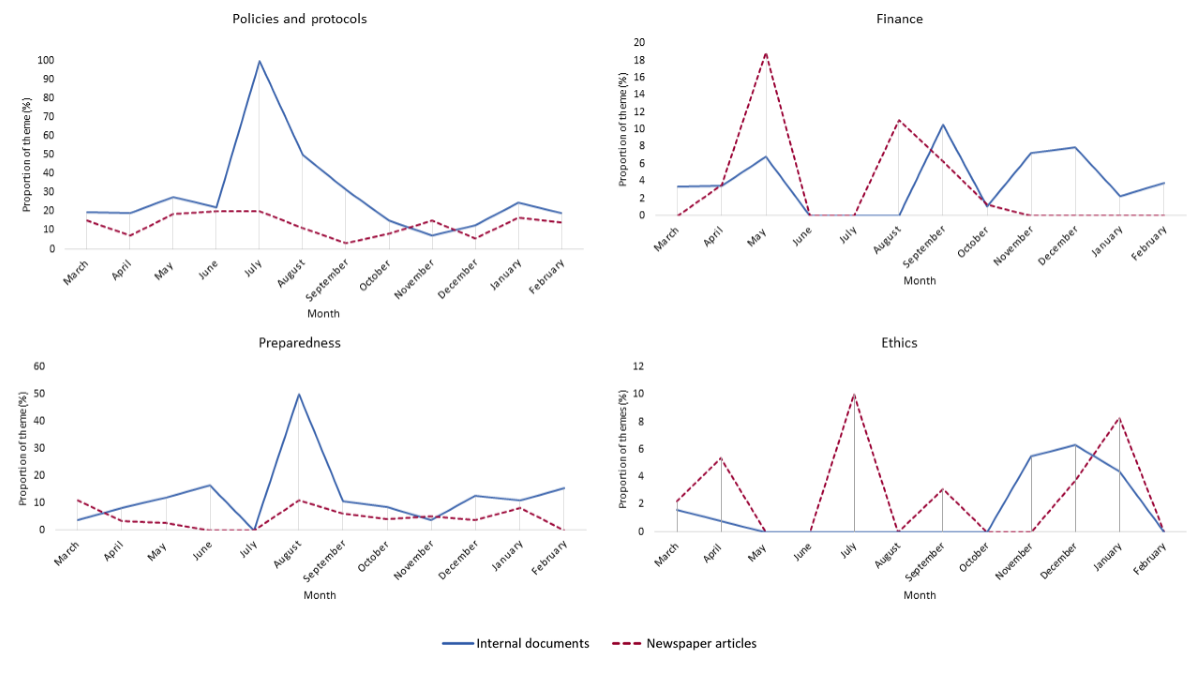
The distribution of theme proportions per month for the hospital documents and newspaper articles during the first year of the COVID-19 pandemic in the Netherlands (March 1, 2020, to February 28, 2021).

During the first year of the pandemic, the attention toward themes in both the internal documents and newspaper articles fluctuated and showed both similarities and differences (Figures 1-3). During the first wave of the pandemic (March to May 2020), the attention given to the themes was largely similar. The content of both the internal documents and newspaper articles mainly related to COVID-19 and regular care capacity, the change in policies and protocols, forms of collaboration to accommodate the large influx of patients with COVID-19, and human resources needed to handle the crisis.

Between July and September 2020, some differences in attention toward themes arose. Specifically, the internal documents mainly covered the themes of policies and protocols and preparedness. The documents revealed that the hospitals reflected on the experiences of the first wave and the lessons learned. This was done to be able to better accommodate a potentially expected new wave after the summer, whereby policies and protocols were adjusted accordingly. The hospitals seemed to have had difficulty in making decisions on certain issues, given the uncertainty of a potential new wave and the lack of clarity on the effectiveness of certain policies. For example, the internal documents contained discussions on whether policies and protocols should be adjusted for staff and patients coming from countries with a high prevalence of infections. Suggestions were made to impose a 2-week quarantine for individuals returning from holidays in these countries. Ultimately, it was decided to follow the national recommendations. The newspaper reported on the themes of policies and protocols and preparedness at a more aggregate level. Instead, the articles focused largely on regular care capacity. The articles described the large backlash of regular care that occurred owing to most care practices being canceled during the first wave. Moreover, the articles described how hospitals aimed to catch up with regular care and the difficulties associated with that owing to, for example, staff being on holidays.

As depicted in Figure 2, a large proportion of the newspaper articles published between July and September 2020 also focused on staff well-being. This was ahead of the attention given to that theme in the internal documents. The newspaper articles described the physical and emotional impacts of the first wave on hospital staff, the insufficiency of a 2-week holiday for full recovery, and staff concerns regarding a potential new wave after the summer period. Similarly, public support became a focal point in the newspaper articles from August onward, before receiving attention in the internal documents. The articles highlighted the decline in public support after the first wave, with the hospital staff increasingly experiencing (verbal) aggression. Moreover, the articles reported on government plans to offer a bonus to health care workers as a sign of appreciation and the ensuing backlash owing to strict eligibility criteria.

From October 2020 onward, the attention toward themes largely aligned again. The main exception was coverage of the themes well-being and public support during January and February 2021, when the third wave of admissions of patients with COVID-19 declined. During this period, the internal documents frequently covered the well-being of staff. The documents showed that the hospitals sought ways to show appreciation and deemed it important to pay attention to the mental well-being of staff, as the work started to take its toll. Instead, the newspaper articles increasingly focused on public support. The articles described the growing tension in society, whereby skeptics, for example, called hospitals to check whether intensive care units were indeed occupied with patients with COVID-19. Furthermore, the newspaper articles described how hospital staff had to deal with increasing aggression.

### Types of Articles

We distinguished 3 types of newspaper articles (refer to Table 2 for an overview of the types and number of newspaper articles). The first type regarded articles that included stakeholders as a source in the reporting. This included, for example, interviews with the chief executive officers or other types of hospital staff or reporting on specific issues such as having to deal with a large influx of patients with COVID-19 and the perspectives of stakeholders on this. The second type regarded articles that did not use stakeholders as a source or where this at least was not stated. This mainly entailed more general reporting, such as the use of eHealth services or changes in hospital visitor policies. As a third type, the newspaper published diary articles in which staff from the hospitals wrote about their personal experiences of working during the crisis. Staff, for example, wrote about the struggle of having to deal with increased emotional and work pressure whereas part of the general public believed COVID-19 to be comparable with the flu. The theme well-being was covered predominantly in diary articles in the newspaper (10/27, 37%).

**Table 2:** The types and number of articles published in the newspaper related to the hospitals’ situation during the first year of the COVID-19 pandemic (March 1, 2020, to February 28, 2021).

Type of newspaper article	Values (N=194), n (%)
Article including hospital as a source	132 (68)
Article not including hospital as a source	39 (20.1)
Diary article	23 (11.9)

### Comparison of Content

All the newspaper articles were assessed for the presence of misinformation. For 7 (3.6%) of the 194 newspaper articles, the content could not be compared with the content of the internal documents because the internal documents did not contain related information. This was, for example, the case for an article published on April 4, 2020, stating that the hospitals in the studied province had the most hospital admissions in the entire country. This could not be verified, as the internal documents did not contain data on admissions in other regions. For all other newspaper articles (187/194, 96.4%), the content could be compared with the internal documents. No indication of the presence of misinformation was found. Multimedia Appendix 2 provides an example for every theme of information described in the newspaper compared with the internal documents.

## Discussion

### Principal Findings

This study aimed to examine newspaper coverage of the situation in hospitals in the Netherlands during the initial 12 months of the COVID-19 pandemic. Previous research found that hospital information was frequently included in media reporting during the COVID-19 pandemic [8,29]. However, our study specifically examined how hospitals navigated the first year of the pandemic and how this reality was communicated to the public through (web-based) newspaper articles. In total, we identified 12 themes that characterized the situation. We observed significant differences in the extent of coverage between internal organizational documents and newspaper articles for 5 (42%) of these 12 themes, namely COVID-19 capacity, regular care capacity, public support, material resources, and policies and protocols. An analysis of changes over time mainly revealed discrepancies in the attention paid to these themes between the first and second waves (May to September 2020) and at the end of the third wave (January and February 2021). Three types of articles could be distinguished, namely those that included stakeholders as a source, those that did not include stakeholders as a source, and diary articles. No indication of the presence of misinformation was found in any of the newspaper articles.

The discrepancies between newspapers and hospitals in their focus on themes can be attributed to their gatekeeping roles [59,60]. Media are faced with a plethora of events that could be covered and, therefore, have to decide which events will eventually be reported on and how [60]. During this process, several factors are considered, including timeliness, importance, proximity, and novelty [60]. Regarding our findings, the consideration of importance for readers (ie, the general public) could explain why newspaper articles covered significantly more about COVID-19 capacity, regular care capacity, and public support. Especially during the summer of 2020, the focus on regular care capacity in the newspaper deviated from the hospitals’ focus on policies and protocols and preparedness. Considering the growing need for regular care to be scaled up again after being largely canceled during the first wave [28], it could be that the newspaper deemed it necessary to frequently report on this issue, given the implications it had for the general population. The newspaper’s increased focus on public support from September 2020 onward may have been driven by societal debates on the appropriateness of public health measures and the related decrease in public support over time [69,70]. Moreover, the lower emphasis on policies and protocols and material resources could reflect the newspaper’s perception that these topics were of less importance to the readers, as they mainly had implications for hospitals and staff. However, the role of hospitals as frontline gatekeepers could also have contributed to this disparity [59,60]. As the primary source of information, organizations can decide which information is being shared with the media [60]. The hospitals may have provided less information regarding material resources and changes in policies and protocols because they deemed it irrelevant or wanted to keep it private.

When examining the attention toward certain themes over time, our findings support the notion that the flow of information can be considered multidirectional. Previous studies have shown how, especially owing to social media, public health communication currently occurs in an arena where public health actors, the public, and news media are coproducing and responding to messaging [2,71]. In contrast to the more unidirectional flow of information, where public health actors disseminate information to the public through media, the current context involves a greater exchange of information between these entities. Studies have, for example, shown how public health actors monitor (social) media to assess the response to and the potential effectiveness of communication [2,71]. Our results suggest that the multidirectional nature of information should also be considered for hospitals. That is, not only do hospitals form an important source of information to promptly inform the public about recent developments, but newspaper articles can also yield relevant information for hospitals. For example, the themes well-being and public support gained attention in newspaper articles before gaining attention in the internal documents of hospitals, which shows that newspapers can be a means for those leading the crisis response to become aware of issues that might affect the organization. Moreover, the type of information found in articles, including details on available capacity, absenteeism rates, and experiences of frontline staff on working during the crisis, can both meet the information needs of staff [72] and help those leading the crisis to assess and compare their internal situation with the situation of others. Although the extent to which newspapers provide new information and act as agenda setters might vary depending on the context [41], our results thus suggest that taking stock of media reporting can be valuable for hospital staff, and organizational leaders in particular, to be aware of changes occurring in both the external and internal environments and how these might impact the organization.

Contrary to what the public or stakeholders might perceive [5,6,73] and what some studies during prior health crises found [37,74], we did not find any indication of the presence of misinformation in any of the newspaper articles. Therefore, in line with previous research, our findings suggest that newspapers can be a credible source for those seeking valid information during a health crisis [35,36]. One explanation for not finding misinformation compared with previous studies could be that the study focused on hospital information instead of risk information, the latter being potentially characterized by more uncertainty [18,37]. Another explanation is that the studied newspaper was a regional newspaper. Previous studies found that compared with national newspapers, regional newspapers tend to have higher quality in reporting a local event because of audience considerations (ie, a local audience prefers more details of a local event than national audiences) and better access to local sources [75,76]. Given the frequent inclusion of local sources, including diary articles written by hospital staff, our findings seem to support these studies. The large inclusion of sources supports the notion that during health crises, health systems would benefit from good relationships between stakeholders and traditional media [42,73,74].

### Practical Implications

Our findings have several implications for crisis communication and health emergency preparedness. First, in light of the ongoing debate surrounding fake news and media distrust, our findings suggest that newspapers can be a valuable source of valid information during health crises [35,36]. Second, health care organizations should reflect upon and be aware of their role in informing the public and how this can be done through the media. Furthermore, health care organizations should be aware that newspapers can provide valuable information to them, allowing them to anticipate the developments occurring in both their external and internal environments. For newspapers, the inclusion of sources forms an important aspect for providing coverage that is reflective of organizational reality [76]. The use of nontraditional forms of reporting, such as diary articles, can be valuable in this regard. Furthermore, using a variety of reporting styles enables newspapers to cater to a diverse audience. The general public, as recipients of news, encompasses groups with diverse characteristics, including differences in sex, education level, and socioeconomic status [77]. These differences can influence the perceived relevance of information and subsequent behavioral responses [78]. By diversifying communications toward different groups of recipients, the effectiveness of communications can be enhanced [77]. Furthermore, newspapers need to be aware of their gatekeeping role, as this can explain deviances in the attention given to certain aspects. Although relevance to readers can justify these deviances, a decreased focus on relevance could make reporting more reflective of organizational reality. Ultimately, the public benefits from health organizations and the media collaborating in crisis communication [42,73]. By fostering relationships before a crisis occurs, both parties can ensure the rapid spread of valid information to the public [42,73].

### Limitations

This study is subject to several limitations. First, the hospital theme proportions were constructed based on the content found in the action and decision lists. These sources reflect the outcome of a larger process, whereby it could be that verbally or via other means, such as email, more attention toward certain themes was given than was observed in the action and decision lists. Therefore, the actual focus of hospitals on themes throughout the year could differ from what we identified through our analysis. Nevertheless, considering the extensiveness of these documents (763 pages from 5 hospitals) and that these documents captured all aspects on which the hospitals needed to decide or take action, the focus derived from the action and decision lists would likely reflect the overall attention to a large extent. A second limitation regards the fact that in our study, we focused on a regional print newspaper, which could differ in reporting from national newspapers and other types of traditional media, including (local) radio and television. However, for local issues, the local newspaper can often be the agenda setter that other media follow, meaning that how the local newspaper reports on an issue can influence how it is presented in other media [76]. Moreover, considering that a substantial part of the population still accesses local newspapers as a source of information [44], studying this type of media itself could be considered still of value to health communication research.

### Conclusions

The COVID-19 pandemic constituted a health crisis in which hospitals were a main actor and source of information. The situation of hospitals could be categorized into 12 themes, and there was a significant difference in focus between the internal hospital documents and newspaper articles for 5 (42%) of the 12 identified themes. Newspaper articles focused significantly more on COVID-19 capacity, regular care capacity, and public support and significantly less on material resources and policies and protocols. The deviances seem to have mainly occurred between the first and the second COVID-19 waves and at the end of the third wave. The gatekeeping roles of the media and stakeholders could explain the deviances in focus. Some themes gained attention in the newspaper articles before gaining attention in the internal hospital data, supporting the notion that the flow of information can be multidirectional. Regarding newspaper reporting on hospital reality, no indication of the presence of misinformation was found. Overall, our findings suggest that newspapers can be a valuable source of information for both the public and health organizations during a health crisis.

## Appendix

Multimedia Appendix 1 Codebook.

Multimedia Appendix 2 Comparison of the information found in the newspaper articles with that found in the internal documents of the 5 Dutch hospitals.

## Data Availability

The data sets generated and analyzed during this study are available from the corresponding author upon reasonable request.

## References

[1] Lu X, Jin Y, Coombs T, Holladay SJ (2022). Integrating strategy and dosage: a new conceptual formula for overcoming unintended effects in public health crisis communication (PHCC). The Handbook of Crisis Communication.

[2] MacKay M, Colangeli T, Gillis D, McWhirter J, Papadopoulos A (2021). Examining social media crisis communication during early COVID-19 from public health and news media for quality, content, and corresponding public sentiment. Int J Environ Res Public Health.

[3] Glik DC (2007). Risk communication for public health emergencies. Annu Rev Public Health.

[4] Barry JM (2009). Pandemics: avoiding the mistakes of 1918. Nature.

[5] Piltch-Loeb R, Savoia E, Goldberg B, Hughes B, Verhey T, Kayyem J, Miller-Idriss C, Testa M (2021). Examining the effect of information channel on COVID-19 vaccine acceptance. PLoS One.

[6] Fernández-Torres MJ, Almansa-Martínez A, Chamizo-Sánchez R (2021). Infodemic and fake news in Spain during the COVID-19 pandemic. Int J Environ Res Public Health.

[7] Kiousis S (2001). Public trust or mistrust? Perceptions of media credibility in the information age. Mass Commun Soc.

[8] Mach KJ, Salas Reyes R, Pentz B, Taylor J, Costa CA, Cruz SG, Thomas KE, Arnott JC, Donald R, Jagannathan K, Kirchhoff CJ, Rosella LC, Klenk N (2021). News media coverage of COVID-19 public health and policy information. Humanit Soc Sci Commun.

[9] Rossmann C, Meyer L, Schulz PJ (2018). The mediated amplification of a crisis: communicating the A/H1N1 pandemic in press releases and press coverage in Europe. Risk Anal.

[10] Dudo AD, Dahlstrom MF, Brossard D (2016). Reporting a potential pandemic: a risk-related assessment of avian influenza coverage in US newspapers. Sci Commun.

[11] Lee ST, Basnyat I (2013). From press release to news: mapping the framing of the 2009 H1N1 A influenza pandemic. Health Commun.

[12] Melki J, Tamim H, Hadid D, Farhat S, Makki M, Ghandour L, Hitti E (2022). Media exposure and health behavior during pandemics: the mediating effect of perceived knowledge and fear on compliance with COVID-19 prevention measures. Health Commun.

[13] Zhang L, Kong Y, Chang H (2015). Media use and health behavior in H1N1 flu crisis: the mediating role of perceived knowledge and fear. Atl J Commun.

[14] Bekalu MA, Dhawan D, McCloud R, Pinnamaneni R, Viswanath K (2021). Adherence to COVID-19 mitigation measures among American adults: the need for consistent and unified messaging. Health Educ Res.

[15] Dhawan D, Bekalu M, Pinnamaneni R, McCloud R, Viswanath K (2021). COVID-19 news and misinformation: do they matter for public health prevention?. J Health Commun.

[16] Romer D, Jamieson KH (2021). Conspiratorial thinking, selective exposure to conservative media, and response to COVID-19 in the US. Soc Sci Med.

[17] Mheidly N, Fares J (2020). Leveraging media and health communication strategies to overcome the COVID-19 infodemic. J Public Health Policy.

[18] Klemm C, Das E, Hartmann T (2014). Swine flu and hype: a systematic review of media dramatization of the H1N1 influenza pandemic. J Risk Res.

[19] Généreux M, David MD, O'Sullivan T, Carignan MÈ, Blouin-Genest G, Champagne-Poirier O, Champagne É, Burlone N, Qadar Z, Herbosa T, Hung K, Ribeiro-Alves G, Arruda H, Michel P, Law R, Poirier A, Murray V, Chan E, Roy M (2021). Communication strategies and media discourses in the age of COVID-19: an urgent need for action. Health Promot Int.

[20] Garfin DR, Silver RC, Holman EA (2020). The novel coronavirus (COVID-2019) outbreak: amplification of public health consequences by media exposure. Health Psychol.

[21] Gesser-Edelsburg A, Stolero N, Mordini E, Billingsley M, James JJ, Green MS (2015). Emerging infectious disease (EID) communication during the 2009 H1N1 influenza outbreak: literature review (2009-2013) of the methodology used for EID communication analysis. Disaster Med Public Health Prep.

[22] Koinig I, Sarfraz M, Ivascu L (2021). Risk communication in the age of COVID-19. Risk Management.

[23] Manganello J, Bleakley A, Schumacher P (2020). Pandemics and PSAs: rapidly changing information in a new media landscape. Health Commun.

[24] Tangcharoensathien V, Calleja N, Nguyen T, Purnat T, D'Agostino M, Garcia-Saiso S, Landry M, Rashidian A, Hamilton C, AbdAllah A, Ghiga I, Hill A, Hougendobler D, van Andel J, Nunn M, Brooks I, Sacco PL, De Domenico M, Mai P, Gruzd A, Alaphilippe A, Briand S (2020). Framework for managing the COVID-19 infodemic: methods and results of an online, crowdsourced WHO technical consultation. J Med Internet Res.

[25] Strömbäck J, Tsfati Y, Boomgaarden H, Damstra A, Lindgren E, Vliegenthart R, Lindholm T (2020). News media trust and its impact on media use: toward a framework for future research. Ann Int Commun Assoc.

[26] Alderwick H, Dunn P, Dixon J (2020). England's health policy response to covid-19. BMJ.

[27] Martin BI, Brodke DS, Wilson FA, Chaiyakunapruk N, Nelson RE (2021). The impact of halting elective admissions in anticipation of a demand surge due to the coronavirus pandemic (COVID-19). Med Care.

[28] Wallenburg I, Helderman JK, Jeurissen P, Bal R (2022). Unmasking a health care system: the Dutch policy response to the COVID-19 crisis. Health Econ Policy Law.

[29] Tejedor S, Cervi L, Tusa F, Portales M, Zabotina M (2020). Information on the COVID-19 pandemic in daily newspapers' front pages: case study of Spain and Italy. Int J Environ Res Public Health.

[30] Bruxvoort KJ, Sy LS, Qian L, Ackerson BK, Luo Y, Lee G, Tian Y, Florea A, Aragones M, Tubert JE, Takhar HS, Ku JH, Paila YD, Talarico CA, Tseng HF (2021). Effectiveness of mRNA-1273 against delta, mu, and other emerging variants of SARS-CoV-2: test negative case-control study. BMJ.

[31] Nyberg T, Ferguson NM, Nash SG, Webster HH, Flaxman S, Andrews N, Hinsley W, Bernal JL, Kall M, Bhatt S, Blomquist P, Zaidi A, Volz E, Aziz NA, Harman K, Funk S, Abbott S, Hope R, Charlett A, Chand M, Ghani AC, Seaman SR, Dabrera G, De Angelis D, Presanis AM, Thelwall S (2022). Comparative analysis of the risks of hospitalisation and death associated with SARS-CoV-2 omicron (B.1.1.529) and delta (B.1.617.2) variants in England: a cohort study. Lancet.

[32] Yang Y, Li Y, An Y, Zhao YJ, Zhang L, Cheung T, Hall BJ, Ungvari GS, An FR, Xiang YT (2021). Workplace violence against chinese frontline clinicians during the COVID-19 pandemic and its associations with demographic and clinical characteristics and quality of life: a structural equation modeling investigation. Front Psychiatry.

[33] de Las Heras-Pedrosa C, Jambrino-Maldonado C, Rando-Cueto D, Iglesias-Sánchez PP (2022). COVID-19 study on scientific articles in health communication: a science mapping analysis in web of science. Int J Environ Res Public Health.

[34] Berry TR, Wharf-Higgins J, Naylor PJ (2007). SARS wars: an examination of the quantity and construction of health information in the news media. Health Commun.

[35] Hilton S, Hunt K (2011). UK newspapers' representations of the 2009-10 outbreak of swine flu: one health scare not over-hyped by the media?. J Epidemiol Community Health.

[36] Duncan B (2009). How the media reported the first days of the pandemic (H1N1) 2009: results of EU-wide media analysis. Euro Surveill.

[37] Birnbrauer K, Frohlich DO, Treise D (2017). Inconsistencies in reporting risk information: a pilot analysis of online news coverage of West Nile Virus. Glob Health Promot.

[38] Husemann S, Fischer F (2015). Content analysis of press coverage during the H1N1 influenza pandemic in Germany 2009-2010. BMC Public Health.

[39] Vasterman PL, Ruigrok N (2013). Pandemic alarm in the Dutch media: media coverage of the 2009 influenza A (H1N1) pandemic and the role of the expert sources. Eur J Commun.

[40] Hubner A (2021). How did we get here? A framing and source analysis of early COVID-19 media coverage. Commun Res Rep.

[41] Liu Q, Zheng Z, Zheng J, Chen Q, Liu G, Chen S, Chu B, Zhu H, Akinwunmi B, Huang J, Zhang CJ, Ming WK (2020). Health communication through news media during the early stage of the COVID-19 outbreak in China: digital topic modeling approach. J Med Internet Res.

[42] Berg SH, O'Hara JK, Shortt MT, Thune H, Brønnick KK, Lungu DA, Røislien JO, Wiig S (2021). Health authorities' health risk communication with the public during pandemics: a rapid scoping review. BMC Public Health.

[43] Khan Y, Fazli G, Henry B, de Villa E, Tsamis C, Grant M, Schwartz B (2015). The evidence base of primary research in public health emergency preparedness: a scoping review and stakeholder consultation. BMC Public Health.

[44] Newman N, Fletcher R, Robertson C, Eddy K, Nielsen R Reuters institute digital news report 2022. Reuters Institute for the Study of Journalism.

[45] Ziekenhuizen in beeld. Rijksoverheid.

[46] Sterfte. Rijksoverheid.

[47] Hoogste coronasterfte tijdens de eerste golf in zuiden van Nederland. Centraal Bureau voor de Statistiek.

[48] Regionale kerncijfers Nederland Internet. Centraal Bureau voor de Statistiek.

[49] Positief geteste mensen. Rijksoverheid.

[50] Positief geteste mensen in Limburg-Noord Internet. Rijksoverheid.

[51] Positief geteste mensen in Zuid-Limburg Internet. Rijksoverheid.

[52] Sterfte in Limburg-Noord. Rijksoverheid.

[53] Sterfte in Zuid-Limburg. Rijksoverheid.

[54] Over NAZL. Netwerk Acute Zorg Limburg.

[55] (2017). Dagbladen in 2017. Commissariaat voor de Media.

[56] Gedragscode. De Limburger.

[57] Cillekens C (2021). 175 jaar Limburgse kranten: een duik in de historie. Limburg.

[58] Coronavirus - updates tot juni 2020. GGD Zuid Limburg.

[59] Shoemaker PJ, Vos TP (2009). Gatekeeping Theory.

[60] Shoemaker PJ, Vos TP, Stacks DW, Salwen MB, Eichhorn KC (2014). Media gatekeeping. An Integrated Approach to Communication Theory and Research. 2nd edition.

[61] Macnamara J (2005). Media content analysis: its uses, benefits and best practice methodology. Asia-Pacific Public Relations J.

[62] Hsieh H, Shannon SE (2005). Three approaches to qualitative content analysis. Qual Health Res.

[63] Vogler D, Meissner F, Coombs W, Holladay S (2022). Tackling the information overload: using automated content analysis for crisis communication research. Handb Cris Commun West Sussex.

[64] Bowen GA (2009). Document analysis as a qualitative research method. Qual Res J.

[65] Fereday J, Muir-Cochrane E (2016). Demonstrating rigor using thematic analysis: a hybrid approach of inductive and deductive coding and theme development. Int J Qual Methods.

[66] Gifford R, Fleuren B, van de Baan F, Ruwaard D, Poesen L, Zijlstra F, Westra D (2022). To uncertainty and beyond: identifying the capabilities needed by hospitals to function in dynamic environments. Med Care Res Rev.

[67] Gifford RE, van de Baan FC, Westra D, Ruwaard D, Zijlstra FR, Poesen LT, Fleuren BP (2022). There and back again. Examining the development of employee commitment during a prolonged crisis. SSM Qual Res Health.

[68] Graneheim UH, Lindgren BM, Lundman B (2017). Methodological challenges in qualitative content analysis: a discussion paper. Nurse Educ Today.

[69] Resultaten 21e ronde: Draagvlak. Rijksinstituut voor Volksgezondheid en Milieu.

[70] Support measures during COVID-19. Eurofound.

[71] Veil SR, Buehner T, Palenchar MJ (2011). A work‐in‐process literature review: incorporating social media in risk and crisis communication. J contingencies Cris Manag.

[72] Billings J, Ching BC, Gkofa V, Greene T, Bloomfield M (2021). Experiences of frontline healthcare workers and their views about support during COVID-19 and previous pandemics: a systematic review and qualitative meta-synthesis. BMC Health Serv Res.

[73] Cloes R, Ahmad A, Reintjes R (2015). Risk communication during the 2009 influenza A (H1N1) pandemic: stakeholder experiences from eight European countries. Disaster Med Public Health Prep.

[74] Roche JP, Muskavitch MA (2016). Limited precision in print media communication of west Nile virus risks. Science Communication.

[75] Jerit J, Zhao Y, Tan M, Wheeler M (2019). Differences between national and local media in news coverage of the Zika Virus. Health Commun.

[76] Holody K, Park S, Zhang X (2013). Racialization of the Virginia tech shootings: a comparison of local and national newspapers. Journal Stud.

[77] Lungu DA, Røislien JO, Wiig S, Shortt MT, Ferrè F, Berg SH, Thune H, Brønnick KK (2021). The role of recipient characteristics in health video communication outcomes: scoping review. J Med Internet Res.

[78] Kreuter MW, Wray RJ (2003). Tailored and targeted health communication: strategies for enhancing information relevance. Am J Health Behav.

