# Supplementary material for: Newspaper Coverage of Hospitals During a Prolonged Health Crisis: Longitudinal Mixed Methods Study
Source: JMIR Public Health Surveill. 2024 Feb 21;10:e48134. doi: 10.2196/48134 (PMC10918547; doi:10.2196/48134)
Supplement: Multimedia Appendix 1 [file publichealth_v10i1e48134_app1.docx]

| **Code** | **Definition** | **Example** |
| --- | --- | --- |
| COVID-19 capacity | How to ensure adequate levels of COVID-19 capacity. This includes the increase or decrease the capacity of COVID-19 care within the hospital based on the influx of patients and the capacity available in the hospital. Another example is the freeing up of beds for COVID-19 patients and the restructuring or redesigning of wards, or freeing up COVID-19 beds and letting wards having their old function. This code also includes the setup of additional COVID-19 capacity at external locations, such as at hotels or a congress center. | - Scaling up ICU beds on recovery.  - Scaling up to phase 1B nursing (emptying C4) - In progress  - Expand corona outpatient ward capacity because it is not possible to meet the demand of employees. Expanding lines and occupancy- CEO requests that IC be scaled down, because other hospitals are now doing this as well. So that the OR can be used again in the future.  - Last Tuesday, the network consultation discussed a central care reporting point and possibly a Corona hotel. This scenario is being worked out.  - Set up an emergency hospital if the Aachen region has no extra capacity and the Supervisory Board agrees; final decision this afternoon.  - Long-term admissions to *congress center* (emergency hospital) has been agreed, correct naming is still being considered |
| Regular care capacity | How to ensure adequate levels of regular care capacity. This includes increasing or decreasing the capacity of regular care within the hospital based on the influx of patients and the capacity available in the hospital. Another example is the reopening of outpatient clinics and providing regular care again, or the closure of outpatient clinics and the cancellation or postponement of care. | - Further scaling up of the clinic on a weekly basis  - CBT agrees with the proposal to restore the acute care department to its original state and put it into regular use  - Scale up non-Covid beds in department A2  - Start reduction OR program by 50% (Day center and (elective) outpatient ORs)  - CEO has decided that all elective admissions will not take place as a precaution  - Modular shift planning elective program; will be discussed in the Polyclinic Heads consultation |
| Regional and (inter)national collaboration | How to collaborate with external stakeholders. This includes information sharing, shared decision making, sharing resources, the transfer of COVID-19 patients due to capacity shortages and other forms of collaboration of a hospital with other hospitals or stakeholders regionally, nationally or internationally. | - Help out *hospital X* with necessary materials. Contact with *CEO* alignment sharing materials.  - Group app ICs region up to *Town X*  to map the situation region-wide - Works and leads to faster exchange of information.  - General practitioners still often incorrectly refer patients to emergency. Sometimes not sick enough. - Extra consultation with general practitioners |
| Human resources | How to ensure enough staff is available for delivering care. This includes recruitment, the redeployment and repurposing of staff, task and role expansion, working longer shifts, cancelling leave of absence, and training. | - CBT agreement with the proposal regarding the extra flexible call for ICU nurses until January 1, 2021. still needs to be coordinated with the employees on the list of flexible workers. An evaluation must be discussed with the Executive Board before January.  - Prescribing medicines by nurse specialists without BIG (certified) is permitted  - Retired healthcare personnel are contacted in order to be able to scale up at a later time. This pool of people can possibly also be used at a later time for supported tasks and not necessarily care tasks. It is also being examined whether and how they can receive a refresher course. |
| Well-being | How to ensure staff well-being. This includes the provision of psychosocial support, practical support such as the provision of a daycare service, and efforts taken to show appreciation. | - To support the staff, it has already been decided to deploy the BOT team. Doctors Without Borders has also offered to support this. This has not yet been discussed in the OT. The BOT team wants to visit the acute departments and corona departments daily, provided they are instructed to do so by OT / BT. BT agrees. In addition, the use of clinical psychologists can be considered. Important decisions are made in the departments with regard to patient care. Support in this is necessary.  - Furhter work out proposal Psymate app by psychosocial team-  - CBT-Agreement: Proposal relaxation area-30-3 realization 27-Mar-CBT-Elaborate and implement proposal relaxation area-30-3 realization  - Due to possible national decision to suspend education -> urgently work out proposal for staff with children  - Record CEO video message for employees for visible signal and express appreciation  - Board of Directors made € 25,000.00 available for staff appreciation |
| Public support | Signs of the public and government to show support and appreciation, from example through gifts and a financial bonus, and issues faced by hospitals revolving around decreased public support later on during the crisis, including frustration and (verbal) aggression by patients and families, and the strict selection of the government regarding who should receive the financial bonus. | - There are many offers from society on various fronts. The communication department is unable to coordinate this sufficiently, given the high workload at the moment.  - The Friends of Hospital foundation would like to make an amount available as a gesture for investment to the employees of the hospital for their efforts during this crisis period.  - agreement: communication campaign #Weeslief starts tomorrow 05.11.2020  - Inquire about Hospital experiences regarding poster campaign desirable and undesirable behavior of patients.  - CEO and *X* agree together on steps to be taken to prevent undesirable behavior from patients.  - The point of how to deal with desirable and undesirable behavior of patients and policy with regard to supervisors will be discussed again in the crisis consultation on 25 February next. |
| Material goods | Relates to issues revolving around ensuring enough material goods. This includes the purchase, use and reuse of materials needed during the crisis, such as personal protective equipment (PPE) and ventilators. | - Sterilizing mouth masks - national ruling follows March 13, then decision to continue collection-  - *Hospital X* indicates new policy: 1 mask per person can be used which you can keep with you.  - Stock of FFP1 masks is dwindling. The healthcare subteam expressed the wish to interpret this. Material supply is becoming a bottleneck.  Inform X that hire of CT scan is agreed upon.  - Perfuser pumps centrally regulated via IT and FB (24/7)-23-03-20 invenetory of pumps within *Hospital X* total 622 pumps. We are now looking to see if we are free to deploy these pumps. Currently Stock 20 at MIT. A counter is ready where these pumps are delivered. To arrange it practically for the coming weeks we put 10 pumps on the HDCU (doctors room) there it is recorded where the pumps go. form is ready there to record the data with id.  - This morning 2 more breathing machines were bought that can probably be delivered this evening |
| The need for innovation | Relates to the implementation of innovations and related purchases to help with care provision. This includes e-health, the purchase of tablets for video calling and the use of cameras to monitor patients. | - Provision of tablets for ED/acute care/C4 (for video calling with medical specialist and home)-1. This will be resolved in consultation with *X* by cameras and Wifi phones. 2. In addition, a number of iphones have been made available for patients. Care inventories number and informs MIT. *X* will feedback the numbers.  - Cameras (patient rooms) partially installed, supplier (SPIE) does not want to start cabling on patient rooms until Monday; acceleration necessary, request support CBT.  - Dashboard: include/display numbers regarding busy ED. |
| Policies and protocols | The need to develop or implement new policies, protocols and guidelines, such as those revolving around testing and visitors; or the change of policies, protocols and guidelines | - Care staff is informed that no one is allowed to go abroad anymore.  - Agreed: current visitor scheme will be maintained. No visitors on COVID wards.  - Distribute "no shaking hands" flyer.  - FB examines possibility of cordoning off desks: distance 1.5 m from patient.  - Employees returning from risk area with complaints to be tested as soon as possible. |
| Finance | Refers to the issues revolving around the financing of costs incurred during the crisis | - Investigate financial consequences of cancellation of travel (if not insured)-has been investigated. framework has been drawn up. budget code created for possible compensation.  - The joint health insurers have sent a letter stating that they are looking into compensation for loss of income due to the corona crisis. Contact point for Limburg is *Health insurer*. Order from CEO BoM to OT to properly identify all extra costs due to the corona virus.  - The way ICU helicopter flights are claimed is being investigated. |
| Preparedness | How to anticipate to the (potential) upcoming situation. This includes the acquiring of information, the development of plans and strategies to prepare for future waves and taking account lessons learned. | - Reflecting on how to deal with a new peak of Covid-19. - Feedback Wednesday, May 13  - Develop proposal "what to do in the event of running out of PPE".  - Consider/determine where to invest what learned from COVID and hold for future.  - CEO has been in contact with Wuhan: there is a medical team willing to speak to us, chat will take place at 09:00 tomorrow morning. Team from hospital will be assembled. |
| Ethics | Relates to ethical issues (potentially) faced and ethical considerations taken during the crisis, including triage (‘code black’), and whether to allow visitors. | - Staff and crisis teams sometimes face difficult choices, seek support through Medical Ethics Committee if needed-*X* contacts MEC then signals to Communications  - Missed in drafted Ethics Framework who will perform triage. Request to *X* to still describe this in the memo. Then the Ethics Framework is released to all staff who will use it. The Ethics Framework will also be included in I-prova. External communication does not take place.  - From OCT-Medical questions regarding work process and recording treatment policy for triage code black: recording at time of admission who does or does not go to ICU. |
