# Supplementary material for: Newspaper Coverage of Hospitals During a Prolonged Health Crisis: Longitudinal Mixed Methods Study
Source: JMIR Public Health Surveill. 2024 Feb 21;10:e48134. doi: 10.2196/48134 (PMC10918547; doi:10.2196/48134)
Supplement: Multimedia Appendix 2 [file publichealth_v10i1e48134_app2.docx]

**Multimedia Appendix 2.** Comparison of the information found in the newspaper articles with that found in the internal documents of the 5 Dutch hospitals.^a^

| Category | Newspaper article quote | Internal document quote |
| --- | --- | --- |
| COVID-19 capacity | “The hospital in X has been split up in four parts. One part for confirmed corona patients, one for those who might have corona, one for those with a weak immune system who need extra protection and one for regular care” (March 20, 2020). | - “Bed house: We have to make space to accommodate the COVID-19 patients and will separate and cluster patient groups: 1) COVID positive/confirmed 2) COVID suspicious (low/high): distinction no longer maintained 3) Vulnerable (immune compromised) 4) Normal patients (not suspicious). Discharge any patient that can be discharged!” (March 15, 2020) |
| Regular care capacity | “Following the example of other hospitals, X is also scaling down regular care. Part of the planned operations are being cancelled. X is making more beds available to enable the influx of corona patients. There are currently eleven patients with the coronavirus in the clinic and three patients in intensive care” (October 28, 2020). | - “Scale up depending on the pressure on the morning of 28-10. Wednesday 29-10 full scale up in accordance with Phase 4 [reducing regular care capacity, increasing COVID-19 capacity]. We may be already getting full Tuesday afternoon because of this. As of 29-10 we will be fully operational. When scaling up, also identify urgent care” (October 22, 2021). |
| Regional national and international collaboration | “The Limburg hospitals consult on a daily basis on available IC capacity and hospitals take over each other’s IC patients when possible and necessary. On a daily basis corona patients are also being transferred to elsewhere in the country” (March 30, 2020). | - “From the ROAZ [Regional Consultation Acute Care] comes the message that the ICs are full but that there is a stable situation at the moment in all hospitals. Transferring patients is going well. X has problems with IC staffing because staff members are sick” (April 8, 2020). |
| Human resources | “Yesterday, there were 68 patients with the corona virus at X. In addition, 38 people are being nursed who may be infected with COVID-19. ‘It has been all hands on deck for months,’ says Van Opdorp. This leads to high absenteeism due to illness. The absenteeism in both hospitals is currently above 10 percent” (January 20, 2021). | - “We fear we might have to reduce the OR [operation room] capacity in X, since there are too few beds, but especially too few staff. Because we see that for both cure and care, absenteeism is rising with a tenth per day to above 10%” (January 18, 2021). |
| Well-being | “Care professional can use the app to measure their mood and stress on a daily basis. This way, the PsyMate maps out the mental fitness of the employee. The app displays a report that the person can view to gain more insight into changes in burden and resilience. The app complements the efforts of the psychosocial team. The PsyMate is a digital tool that is used at the X psychiatry department to measure daily activities, mood and state of mind of people” (May 8, 2020). | - “Work out proposal Psymate App by psychosocial support team” *(*April 7, 2020*).* - “CBT-in principle agrees with roll-out of psymate for staff by psychosocial care team” *(*April 16, 2020*).* - “The use of psymate has been taken up by HR, now live” *(*April 17, 2020*).* |
| Public support | “The list of function groups that should be eligible for a one-time corona bonus, published by the ministry just before last weekend, has caused concern. Not only among nurse anesthetists in various hospitals, but also among other professional groups that have ended up on the ‘no’ list. Maternity nurses, physiotherapists, OR staff; together with dozens of other functions they all fall by the wayside” (September 23, 2020). | - “We notice that the ‘care bonus’ leads to unrest among personnel. [..]We’ll do our very best as board to interpret the scheme as broadly as possible” (September 24, 2020). |
| Ethics | “Doctors are faced with more ethical dilemmas, such as: what if only one IC bed is available and three patients require admission? That is difficult. It is important in such a case that they do not make such a decision alone and that the decision is supported by a team” (April 3, 2020). | - “Discussed yesterday in COVID team. Request to think in advance about setting up a triage team. Criteria are stated in the NVIC [Dutch Association for Intensive Care] pandemic manual in consultation with the Geriatrics and Geriatrics Association. Transfers/difficult decisions about bed occupancy IC should not be made only by intensivists. Follow IC guidelines and consult with COVID team” (March 23, 2020). |
| Material resources | “We purchased face shields for emergency room workers this week. It is a plastic screen that covers your entire face. Not prescribed as standard equipment, but the people themselves indicated that they would feel safer if they had them, so we took that decision” (Chief executive officer of X in De Limburger, April, 8,2020). | - “Staff have a need for extra protection to contain feelings of fear. X argues for maintaining the current course of action. A shield is a wish of many employees, but does not provide extra protection. FFP2 is used for, among other things, aerosol patients (high-risk procedures). Proposal: FFP2 masks for staff treating infected patients, facilitate surgical mouth masks and (personal) shield during triage” (April 1, 2020). |
| Innovation | “Due to the safety measures surrounding corona, many appointments in the hospital cannot continue in the normal way. X is now looking at how and in what way care can be restarted as soon as possible. One of the means that the hospital uses for this is the video call consultation. An appointment at the outpatient clinic in the hospital is replaced by a digital (video call) consultation with the doctor or nurse” (May 6, 2020). | - “Organizing outpatient telephone consultation hours (video calling). In case of phase red, outpatient clinics will be scaled down and switched video calling. X will join the care team from March 17 2020 onwards, with regard to video calling, triages and tent design. From March 17, a start can be made with video calling. Instruction will be made on how to perform video calling” (March 17, 2020). |
| Policies and protocols | “Hospitals have been limiting the number of visitors a patient can receive for a few days now. At VieCuri and Laurentius, only one visitor is allowed to visit a patient. At MUMC+ and Zuyderland, each patient is only allowed one visitor per day. ‘It is a friendly, but urgent request,’ says the spokesperson for the Maastricht hospital. We appeal to people, to take their responsibility. We will certainly not enforce it strictly, we understand that individual situations can be that people still want to go to father or mother as a couple” (March 13, 2020). | - “Work out proposal regarding restriction of visitors and share with CBT” (March 8, 2020). - “Proposal restriction visitors to CBT [crisis decision team]” (March 9, 2020). - *“*Communicating: urgent request regarding visitor reduction (from now on 1 visitor per patient per day)” (March 10, 2020). |
| Finance | “A lot of money goes to corona patients and the setting up of extra intensive care units, while hardly any money comes in because the planable care has been postponed. From 1 May, hospitals can receive a monthly advance from the health insurers ‘to give them the space to cope in times of crisis. comply with their payment relief.’ NVZ [the Dutch Hospital Association] leader Ad Melkert acknowledges that it has been ‘very dire’ for the hospitals in recent weeks” (April 22, 2020). | - “There is consultation with VWS [the ministry of health], NVZ [the Dutch Hospital Association], NFU [the Dutch Academic Hospital Association] and Zorgverzekeraar Nederland [the Dutch Health Insurers Association] in which they will look at how the hospitals are doing, how the liquidity of the hospitals is. After corona one can expect a lot of backlog. X receives an assignment together with 1 cutter, 1 viewer, 1 manager and HRM how to deal with this/how to update it” (March 25, 2020). |
| Preparedness | “In order to be well prepared for an even greater influx of corona patients, hospital X will expand its capacity toward the conference center next week. In one of the halls there will be 250 beds in the next few days. A hospital spokesperson confirmed this. The emergency shelter should be operational in the course of next week. The care in the conference center is intended for patients who have been diagnosed with the coronavirus, but who are not eligible for IC care and for whom basic care is sufficient. This concerns low-complex, nursing care. The decision to use the conference center as an ‘overflow’ for the hospital was taken yesterday afternoon. Whether the extra capacity will actually be used depends on how the corona epidemic will develop” (March 27, 2020). | - “For X: difference between our emergency hospital and the other one (describe care well or call it no care at all, better cure with a maximum of treatment), + financial picture + mapping out risks. X asks to make proposal for processes in emergency hospital” (March 25, 2020). - “Long-term care X (emergency hospital) has been agreed, correct naming is still being considered” (March 26, 2020). |

^a^ Where necessary, the quotes have been edited to preserve anonymity.
